# Zoo gut plastispheres enable pathogen escape and adaptation

**DOI:** 10.1093/ismejo/wrag207

**Published:** 2026-08-05

**Authors:** Zipei Luo, Yilun Liu, Haimei Wu, Yunmu Xiao, Yong Li, Meizhi Liu, Changchao Li, Dong Zhu, Ling N Jin, Tao Dong, Wende Yan

**Affiliations:** National Engineering Laboratory of Applied Technology for Forestry & Ecology in South China, and Laboratory of Urban Forest Ecology of Hunan Province; Department of Ecology and Environment, Central South University of Forestry and Technology, Changsha, Hunan Province 410004, China; National Engineering Laboratory of Applied Technology for Forestry & Ecology in South China, and Laboratory of Urban Forest Ecology of Hunan Province; Department of Ecology and Environment, Central South University of Forestry and Technology, Changsha, Hunan Province 410004, China; National Engineering Laboratory of Applied Technology for Forestry & Ecology in South China, and Laboratory of Urban Forest Ecology of Hunan Province; Department of Ecology and Environment, Central South University of Forestry and Technology, Changsha, Hunan Province 410004, China; National Engineering Laboratory of Applied Technology for Forestry & Ecology in South China, and Laboratory of Urban Forest Ecology of Hunan Province; Department of Ecology and Environment, Central South University of Forestry and Technology, Changsha, Hunan Province 410004, China; Key Laboratory of Food & Environment & Drug Monitoring and Testing of Universities in Hunan Province, Hunan Police Academy, Changsha 410138, China; National Engineering Laboratory of Applied Technology for Forestry & Ecology in South China, and Laboratory of Urban Forest Ecology of Hunan Province; Department of Ecology and Environment, Central South University of Forestry and Technology, Changsha, Hunan Province 410004, China; National Engineering Laboratory of Applied Technology for Forestry & Ecology in South China, and Laboratory of Urban Forest Ecology of Hunan Province; Department of Ecology and Environment, Central South University of Forestry and Technology, Changsha, Hunan Province 410004, China; Department of Civil and Environmental Engineering, The Hong Kong Polytechnic University, Kowloon, Hong Kong 999077, China; State Key Laboratory of Regional and Urban Ecology, Ningbo Observation and Research Station, Institute of Urban Environment, Chinese Academy of Sciences, Xiamen 361021, China; Zhejiang Key Laboratory of Pollution Control for Port-Petrochemical Industry, CAS Haixi Industrial Technology Innovation Center in Beilun, Ningbo 315830, China; Department of Civil and Environmental Engineering, The Hong Kong Polytechnic University, Kowloon, Hong Kong 999077, China; Department of Health Technology and Informatics; Research Centre for Nature-based Urban Infrastructure Solutions; Research Centre for Future Food; Mental Health Research Centre, The Hong Kong Polytechnic University, Kowloon, Hong Kong 999077, China; State Key Laboratory of Marine Environmental Health, City University of Hong Kong, Kowloon, Hong Kong 999077, China; Department of Immunology and Microbiology, School of Life Sciences, Southern University of Science and Technology, Shenzhen 518055, China; National Engineering Laboratory of Applied Technology for Forestry & Ecology in South China, and Laboratory of Urban Forest Ecology of Hunan Province; Department of Ecology and Environment, Central South University of Forestry and Technology, Changsha, Hunan Province 410004, China

**Keywords:** plastisphere, pathogen adaptation, antibiotic resistance genes (ARGs), zoo gut microbiome

## Abstract

In zoos, intensive human contact and artificial feeding may create pathways for microplastic (MP) ingestion and gut colonization. We hypothesized that ingested MPs form intestinal plastispheres with elevated pathogenic potential and enhanced environmental persistence. To test this, we surveyed feces from 15 zoo-dwelling species and coupled particle characterization, feces-derived intestinal simulations, metagenomic sequencing, and a subsequent water-exposure experiment. Zoo feces contained more abundant MPs than reported for wild counterparts, with fragments predominating and polyethylene terephthalate (PET)/polystyrene (PS) dominating polymer profiles. MP burdens tracked human–animal interaction patterns, with human-fed species (e.g. Tiger, Elephant) carrying the highest loads (88–212 items/g). MPs supported dense biofilms whose composition diverged from bulk gut communities, exhibiting greater compositional variability and substrate-specific assembly. Metagenomic analyses revealed coordinated enrichment of potentially pathogenic taxa, virulence factor genes (VFGs), and antibiotic resistance genes (ARGs), with ARG profiles dominated by efflux- and inactivation-related mechanisms and tightly associated with mobile genetic elements. Elevated Type II/III/IV/VI secretion systems and effector delivery–related VFGs occurred within extracellular polymeric substance–rich biofilms, suggesting enhanced potential for ARG retention and horizontal gene transfer. During the 35-day aquatic exposure, MP-associated communities persisted longer than non-plastic particle-associated communities and free gut microbiota, suggesting that plastic-specific properties promote microbial persistence. PET/PS plastispheres showed the slowest declines in bacterial activity and favored the persistence of *Enterococcus*, *Enterobacter*, and *Clostridium.* Overall, intestinal MPs in zoo animals may select, enrich, and export high-risk microbiomes, highlighting the need for MP mitigation and evidence-based management of zoos and adjacent ecosystems.

## Introduction

Urbanization intensifies the production, use, and dispersal of persistent organic pollutants [1]. Among them, microplastics (MPs) have emerged as a pervasive concern due to their persistence, ubiquity across food webs, and capacity to accumulate in multiple trophic levels, amplifying biological impacts [2–5]. Owing to their diverse shapes, chemistries, and high surface-area-to-volume ratios, MPs provide abundant attachment sites for microorganisms and support biofilm-based communities collectively termed the “plastisphere”, here defined as the MP surface-associated biofilm microenvironment and its colonizing microorganisms [6–8]. Once incorporated into food chains, MPs may elevate exposure to opportunistic and frank pathogens [9–14], raising risks to ecosystem and human health [15, 16].

Mounting evidence shows that plastispheres selectively enrich taxa and functions not proportionally represented on natural substrates. Plastisphere biofilms can promote the proliferation of specific pathogenic bacteria and act as hubs for the transmission of antibiotic resistance genes (ARGs). For example, plastispheres often harbor higher abundances of potential pathogens than surrounding matrices [17], and low-density polyethylene (PE) MPs can preferentially retain antibiotic-resistant bacteria and ARGs within biofilms [18]. These observations suggest that MPs create refuges that foster pathogen survival, reproduction, and spread. Yet, a critical knowledge gap remains the origins of plastisphere pathogens—particularly those arising from animal intestines—and their subsequent environmental adaptability have rarely been examined, despite their importance for disease transmission and intervention strategies.

Plastispheric pathogens can originate from multiple reservoirs, including soil, water, air, and host-associated microbiomes. Marine studies, for example, have linked plastisphere-borne *Vibrio* pathogens to ambient seawater sources [19], whereas other work has identified human- and plant-associated pathogens exclusively within plastisphere biofilms [20]. Zoo environments offer a unique and underexplored model to bridge these domains. Zoo-dwelling higher terrestrial animals frequently interact with humans and are maintained under artificial feeding regimens, increasing opportunities for MP ingestion and gut colonization. Their intestines host diverse microbiota capable of adhering to plastic surfaces, potentially exporting gut-derived plastispheres into surrounding habitats via fecal pathways. However, the extent to which zoo–human interaction patterns shape intestinal MP burdens, and whether gut-derived plastispheres exhibit enhanced pathogenic potential and environmental persistence, remains unclear.

Here, we conduct a cross-species survey of intestinal MPs in 15 zoo animal species and couple these observations with feces-derived intestinal simulations to characterize plastisphere composition, resistance, and virulence potential. Using complementary particle characterization (stereomicroscopy, FTIR, and Raman), metagenomic sequencing, and a subsequent water-exposure experiment including non-plastic particle and no-particle controls, we assess: (i) how MP characteristics (abundance, size, polymer type) relate to human–animal interaction patterns; (ii) whether microbial communities on MP surfaces differ from bulk gut microbiota; (iii) whether gut-derived plastispheres exhibit enrichment of ARGs, virulence factors, and secretion systems indicative of heightened pathogenicity and horizontal gene transfer; and (iv) whether plastisphere-associated bacteria persist during environmental exposure beyond what can be explained by generic particle-associated biofilm formation, particularly in aquatic settings. We show that intestinal MPs in zoo animals can act as a “haven” that selects for, amplifies, and exports pathogenic and antimicrobial-resistant consortia from hosts to the environment, with implications for pollution control, husbandry practices, and environmental health risk management.

## Materials and methods

### Animal feces sampling strategy and separation of MPs

A total of 15 animal fecal samples were manually collected using a metal spatula and nitrile gloves and placed into sterile 1000 ml glass jars, the exteriors of which were wrapped in aluminum foil. For species with large population sizes, fecal samples were collected from different locations within the enclosure to minimize the likelihood of repeated sampling from the same individual and thereby improve sample representativeness. To reduce the risk of plastic fiber contamination during the field sampling, contact between collection materials and synthetic clothing or other plastic items was carefully avoided. All samples were labeled with the collection date, sample type (feces), and sampling location. To characterize differences in visitor exposure, enclosures were further classified into three human–animal interaction categories according to the degree and mode of visitor contact. The “human feeding interaction” category included enclosures in which visitors were permitted to directly feed animals under zoo supervision. The “no interaction in open enclosures” category included open-display settings where visitors could observe animals without physical barriers but were not allowed to touch or feed them. The “closed observation” category included barrier-separated enclosures where visitors were limited to observation only, with no opportunity for direct contact or feeding. In the latter two categories, animals were fed exclusively by zookeepers during routine husbandry. Therefore, the main distinction among categories was visitor access and feeding opportunity rather than routine feeding management, which was consistent across enclosure types. After collection, all samples were immediately transported to the laboratory, preserved in ethanol, and stored at −20°C until MP separation to minimize degradation of organic matter and potential changes in particle characteristics. Sample collection was authorized by Changsha Ecological Zoo, and zoo staff accompanied the entire sampling process.

The extraction of MPs from fecal samples was based on previously published protocols with minor adaptations [21–23]. In brief, frozen samples were first thawed, and the preservative ethanol was carefully removed to reduce interference during subsequent processing. The fecal material was then transferred to clean metal vessels, and both wet mass and oven-dried mass were recorded. Drying was conducted overnight at 50°C, after which the samples were homogenized into fine powder using a sterile mortar. For chemical digestion, the homogenized material was treated with pre-filtered 10% KOH solution at a solution-to-sample ratio greater than 5:1 and incubated at 50°C for ~12 h to facilitate the breakdown of organic matter [24]. The digested mixtures were subsequently subjected to density separation using 100 ml of pre-filtered hypersaline NaCl solution (density: 1.2 g/ml). After agitation, the suspension was allowed to settle for 10 min, and the supernatant was recovered. This separation step was repeated twice to maximize particle recovery, with rinsing of the vessel walls using Milli-Q water during each round to minimize particle loss. The combined supernatants were vacuum filtered through glass microfiber filters (nominal pore size: 1.2 μm). Filters were labeled, placed in sterile 47 mm Petri dishes, and sealed with parafilm to prevent contamination. To further characterize the fecal matrices, lipid content of the remaining available samples from five animal species was measured using the Soxhlet extraction method according to AOAC procedures [25] and expressed on a dry weight basis. The measured lipid contents were 23.3% for Northeast tiger, 6.7% for Elephant, 9.9% for Sika deer, 8.1% for Alpaca, and 8.4% for kangaroo. We acknowledge that digestion efficiency may differ among fecal matrices [26]. However, to minimize false-positive identification, suspected particles were further verified by FTIR and Raman spectroscopy, and only spectroscopically confirmed synthetic polymers were included in the final dataset. The overall experimental workflow is provided in the Supplementary Material (Fig. S1).

### Microplastic identification and characterization

After sample pretreatment, particles retained on the filters were observed under a stereomicroscope (LV-150 N, Nikon, Japan). ImageJ software was used exclusively for morphometric measurements of particle size and shape at 10× and 100× magnifications [27] and was not used for particle identification or polymer classification. To distinguish suspected MPs from residual organic particles, particles were first visually screened according to commonly used morphological criteria, including a homogeneous appearance, clear particle boundaries, absence of visible cellular or tissue structures, and lack of natural organic textures. Given that alkaline digestion may not completely remove all organic residues, especially in complex fecal matrices, visual screening was used only as a preliminary step. Subsequently, all visually suspected particles were subjected to Fourier transform infrared (FTIR) and/or Raman spectroscopy for polymer confirmation. Only particles that were spectroscopically confirmed as synthetic polymers were included in the final quantitative analysis. Confirmed MPs were then categorized into three size classes (20–50 μm, 50–100 μm, 100–500 μm) and three shape types (fragments, fibers, and films). MP abundance was expressed as the number of items per gram (items/g).

For FTIR analysis, measurements were performed in attenuated total reflection mode over the range of 4000–400 cm^−1^. The acquired spectra were compared with the OMNIC software (v9.2), and a match threshold of ≥80% was used for polymer identification, whereas spectra with lower match values were regarded as tentative assignments and interpreted in combination with characteristic absorption peaks. Raman spectra were obtained using a WITec confocal Raman microscope (Alpha 300RS, Germany) equipped with a 532 nm laser diode (<30 mW) and a 100× objective lens at room temperature (~24°C). Polymer composition was identified by comparison with standard FTIR and Raman spectra of known plastics.

### Intestinal simulation experiment

In the absence of validated gastrointestinal simulation systems for Tiger and Elephant, the Simulator of the Human Intestinal Microbial Ecosystem (SHIME) was used as a surrogate in vitro system to examine microbial colonization on MP surfaces following previously described methods [28–30] (Fig. S2). Each unit consisted of five double-jacketed compartments corresponding to the stomach, small intestine, and the ascending, transverse, and descending colon. The stomach and small-intestine vessels were supplied three times daily with 140 ml of SHIME medium and 60 ml of pancreatic solution, respectively. The SHIME medium contained arabinogalactan (1.0 g/L), pectin (2.0 g/L), xylan (1.0 g/L), starch (4.0 g/L), glucose (0.4 g/L), yeast extract (3.0 g/L), peptone (3.0 g/L), mucin (1.0 g/L), and cysteine (0.5 g/L). The pancreatic solution comprised NaHCO_3_ (12.5 g/L), bile salts (6.0 g/L), and pancreatin (0.9 g/L). Five MP treatments (Baoguan Plastics, Dongguan, China) were established by separately adding polyethylene terephthalate (PET), polystyrene (PS), polyvinyl chloride (PVC), PE, or polypropylene (PP), with 1 g of each polymer added to 200 ml of suspension. The MPs were ground, sieved to ~250 μm, rinsed three times with deionized water, and dried in the dark at room temperature prior to use. All compartments were operated under continuous agitation at 36.5°C and maintained under anoxic conditions by N_2_ flushing. The two SHIME units were inoculated separately with Tiger and Elephant fecal microbiota. A treatment without MPs was included as the control. For inoculum preparation, 10 g of fresh Tiger or Elephant feces was mixed with 200 ml of sterile phosphate-buffered saline (PBS), homogenized in a shaking water bath, allowed to settle undisturbed for 30 min, and the supernatant was collected as the inoculum for subsequent experiments [31]. PBS was prepared by dissolving 1 g of phosphate buffer powder in 1 L of distilled water, adjusting the pH to 7.2, autoclaving at 121°C for 20 min, and storing at 4°C. To exclude the possibility of environmental contamination, a negative control was included in which the same polymer particles were incubated under identical simulated intestinal conditions without fecal microbial inoculation. Additional details on reactor setup and inoculum preparation are available in previous studies [32, 33].

After the culture period, MPs together with the associated bacterial biomass were collected by centrifugation at 4°C and 12 000 × g for 20 min. The recovered material was then gently washed with sterile 0.9% NaCl solution twice to remove loosely suspended microbial cells before subsequent analyses. The collected material was divided into two portions. One portion was used for metagenomic sequencing analysis, and the other was used for bacterial extracellular polymeric substances (EPS) component analysis. EPS was extracted following previously published methods [34]. The polysaccharide fraction of EPS was quantified by the anthrone method [35]. Briefly, anthrone reagent was prepared as 0.1% anthrone in 80% (v/v) H₂SO₄, and glucose solutions (0–100 mg/L) were used as standards. For each assay, 1 ml of EPS extract was mixed with 1 ml of reagent in a digestion tube, heated in a 100°C water bath for 14 min, and then cooled in a 5°C water bath for 5 min. Absorbance was measured at 625 nm using a spectrophotometer. The protein fraction of EPS was determined using the Bradford method [36]. Bradford reagent was prepared by dissolving 0.1 g Coomassie Brilliant Blue in 50 ml of 95% ethanol, followed by the addition of 100 ml of 85% (w/v) H₃PO₄, and then dilution to 1 L with distilled water. Bovine serum albumin (BSA, 0–100 μg/mL) was used to generate the protein standard curve. For analysis, 0.2 ml of EPS extract was mixed with 5 ml of reagent and incubated for 20 min, after which absorbance was recorded at 595 nm. EPS polysaccharides and proteins were quantified from the extracted EPS solution, and the results were expressed as concentrations in the extract (mg/L). In addition, a separate portion of the harvested samples, together with MPs incubated without gut inoculum, were fixed in 2.5% glutaraldehyde, dehydrated through a graded ethanol series, and freeze-dried. These specimens were subsequently examined for cell morphology using scanning electron microscopy (SEM, Quanta F250, Germany).

### Metagenomic sequencing and analysis of samples from the intestinal simulation experiment

To characterize the taxonomic composition and functional potential of microbial communities colonizing MP surfaces in the simulated gut environment, metagenomic sequencing was conducted on 36 samples collected at the end of the intestinal simulation experiment, including 30 MP-associated samples and six corresponding control samples. Specifically, the MP-associated samples included five MP types from the Tiger gut simulation and five MP types from the Elephant gut simulation, each with three biological replicates, whereas the control samples included three biological replicates for each gut simulation. Following incubation, MP particles together with their attached bacterial communities were collected by centrifugation at 12 000 × g and 4°C for 20 min and rinsed twice with sterile 0.9% NaCl to remove loosely attached cells. In the control groups, which did not contain MPs, the gut microbial biomass was first rinsed twice with sterile PBS to remove residual culture medium and then collected by centrifugation under the same conditions. The resulting pellets were immediately stored at −80°C until DNA extraction. Genomic DNA was sheared with a Covaris M220 instrument (Gene Company Limited, China) and fragments with an average length of ~400 bp were recovered for paired-end library preparation using the NEXTFLEX Rapid DNA-Seq kit according to the manufacturer’s instructions. The constructed libraries were sequenced on a NovaSeq 6000 System (Illumina) by Meigene Biotechnology Co., Ltd. (Guangzhou, China). Raw sequencing reads were processed with fastp (v0.23.2) to remove adapters and low-quality sequences [37]. The resulting clean reads were assembled de novo using MEGAHIT (v1.2.9), and only contigs of at least 300 bp were kept for downstream analyses. Open reading frames (ORFs) were predicted with Prodigal (v2.6.3) [38], and predicted ORFs ≥100 bp were translated into amino acid sequences. Redundant sequences were removed by clustering with CD-HIT (v4.8.1) to obtain a nonredundant gene catalog. Gene abundance was calculated by aligning clean reads from each sample against this catalog using SOAPaligner (v2.21), followed by normalization as reads per kilobase per million mapped reads. Detailed statistics for metagenomic sequencing, assembly, and gene prediction are provided in Supplementary Material (Table S2).

Taxonomic annotations were primarily based on the NCBI NR database using DIAMOND (E-value <1 × 10^−5^). In addition, MetaPhlAn (v4.1.1) was used as an independent validation approach for metagenomic taxonomic profiling, and it yielded consistent results at the domain level. Functional annotation was performed separately by aligning representative gene sequences against the Kyoto Encyclopedia of Genes and Genomes (KEGG, download January 2025), Virulence Factor Database (download 31 January 2025), and Comprehensive Antibiotic Resistance Database (v4.0.0) using DIAMOND with the same threshold parameters. Mobile genetic elements (MGEs) were identified by aligning predicted ORFs against local reference databases using BLASTN (BLAST+ v2.14.0), with an E-value cutoff of 1 × 10^−5^, sequence identity >90%, and an alignment length ≥ 50 bp. Raw metagenomic sequencing data generated in this study have been archived in the NCBI Sequence Read Archive under accession number PRJNA1413497.

### Exposure experiment

To investigate the persistence and succession of particle-associated microbial communities after intestinal simulation, a supplementary experiment was conducted using both MP and non-MP particles under the same simulated gut conditions as those used in the metagenomic experiment. Specifically, PET and PS were selected as representative MPs, whereas biochar and silicon dioxide (SiO₂) were used as non-MP particle controls (non-MPs). SiO₂ was selected as an inorganic and relatively stable mineral particle to represent a non-plastic hard surface [39, 40], whereas biochar was selected as a carbonaceous and porous environmental particle with a high capacity to support microbial attachment [41–43]. These non-MP particle controls were used to distinguish MP-specific selection from generic particle-associated biofilm formation. In addition, gut microbial communities incubated without particles served as the particle-free control group. All particles were ~250 μm in size and were added at the same dosage as in the metagenomic intestinal simulation experiment (1 g/200 ml). The same Tiger and Elephant fecal samples used in the original experiment, which had been preserved at −80°C, were used for this supplementary assay. Three biological replicates were included for each treatment. Gut microbial communities collected before particle exposure were used as the baseline group.

Following the intestinal simulation, equal masses of particle samples were transferred into 50 ml sterile centrifuge tubes containing sterile distilled water for a subsequent exposure experiment. The particle-free control consisted of the corresponding gut microbial communities incubated in sterile distilled water without particles. All tubes were maintained under anoxic conditions at 28°C with shaking at 120 rpm to simulate a dynamic aquatic environment and to evaluate microbial persistence on particle surfaces. Samples were collected on Days 0, 7, 14, 21, 28, and 35 during the exposure period. Day 0 represented the particle-associated or particle-free control microbial communities immediately after transfer into sterile distilled water following intestinal simulation. For particle-containing treatments, particles were recovered by centrifugation at 4°C and 12 000 × g for 20 min, and then rinsed twice with sterile 0.9% NaCl solution to remove planktonic cells. For the particle-free control group, microbial cells were collected directly by centrifugation under the same conditions.

Bacterial metabolic activity was determined using a Cell Counting Kit-8 (CCK-8) assay according to the manufacturer’s instructions [44]. After incubation with the CCK-8 reagent, absorbance at 450 nm (OD_450_) was measured using a microplate reader, and the OD_450_ value was used as an indicator of bacterial metabolic activity. In addition, EPS were extracted from each sample, and the contents of major EPS components, including proteins and polysaccharides, were quantified. EPS extraction and the determination of protein and polysaccharide contents were performed using the same procedures described in Section 2.3.

### 16S rRNA gene amplicon sequencing and functional prediction of exposure samples

To characterize bacterial community succession during the exposure experiment, 72 particle-associated samples were subjected to 16S rRNA gene amplicon sequencing. These samples included MP particles (PET and PS) and non-MP particles (biochar and SiO₂) collected from the Tiger and Elephant gut simulations on Days 0, 14, and 35, with three biological replicates for each particle type at each time point. No gut samples were included in this analysis. Microbial DNA was extracted using a DNA extraction kit (MoBio Laboratories Inc., California, USA). PCR amplification of the V3–V4 hypervariable region of the bacterial 16S rRNA gene was performed using a Bio-Rad S1000 thermal cycler (Bio-Rad Laboratories, California, USA) with the universal primer pair 338F (5′-ACTCCTACGGGAGGCAGCA-3′) and 806R (5′-GGACTACHVGGGTWTCTAAT-3′). Sequencing libraries were prepared using the NEBNext Ultra II DNA Library Prep Kit for Illumina (New England Biolabs, Massachusetts, USA) in accordance with the manufacturer’s instructions and index design guidelines. Library quality was assessed using a Qubit 2.0 Fluorometer (Thermo Fisher Scientific, Massachusetts, USA), and sequencing was performed by Guangzhou Meigene Biotechnology Co., Ltd. on a NovaSeq 6000 System (Illumina). Low-quality reads and chimeric sequences were removed, and the remaining high-quality sequences were clustered into operational taxonomic units (OTUs) at 97% sequence similarity. Representative sequences of OTUs were taxonomically assigned against the Greengenes database (v13.8), and an OTU abundance table was generated for downstream microbial community analyses.

Functional prediction of MP-associated and non-MP-associated bacterial communities was performed using PICRUSt (Phylogenetic Investigation of Communities by Reconstruction of Unobserved States, v1.1.4) [45]. Predicted functional profiles were annotated against the KEGG database, and gene functions were classified according to KEGG Orthology. Functional diversity indices were calculated accordingly, and differences in the relative abundance of KEGG pathways at Levels 1 and 3 were used to assess functional variation among samples. The raw sequencing data have been deposited in the NCBI Sequence Read Archive under accession number PRJNA1475087.

### Statistical analysis

Statistical analyses were performed using IBM SPSS Statistics version 22.0 and R version 4.3.0. Data are presented as the mean ± standard deviation. Differences among gut-environment and plastisphere samples were assessed using one-way analysis of variance (ANOVA) or the Kruskal–Wallis test, as appropriate, depending on data distribution and homogeneity of variance. Beta-diversity differences were evaluated by permutational multivariate analysis of variance (PERMANOVA) based on Bray–Curtis dissimilarity. Species niche breadth was estimated using Levins’ niche breadth index implemented in the R package spaa (R, v4.3.0). Community-level niche breadth was calculated as the weighted average of the niche breadth values of all species within each community. Co-occurrence networks among microbial taxa and associations between virulence factor genes (VFGs) and bacterial genera were constructed based on Spearman’s rank correlations |r| > 0.40, with false discovery rate (FDR)-corrected *P* < .05. Network topological properties were analyzed using Gephi (v0.10.1). Differential taxonomic and functional features were identified using the Wilcoxon rank-sum test. Unless otherwise specified, *P* < .05 was considered statistically significant. For multiple comparisons, *P* values were adjusted using the Benjamini–Hochberg FDR and FDR < 0.05 was considered statistically significant. Data visualization was performed using GraphPad Prism (v9.5.0), the Chiplot online platform (https://www.chiplot.online/), BioRender (https://www.biorender.com/), and Microsoft PowerPoint (v16.91).

## Results and discussion

### Human–animal interactions drive high intestinal MP burdens in zoo animals

**Figure 1:**
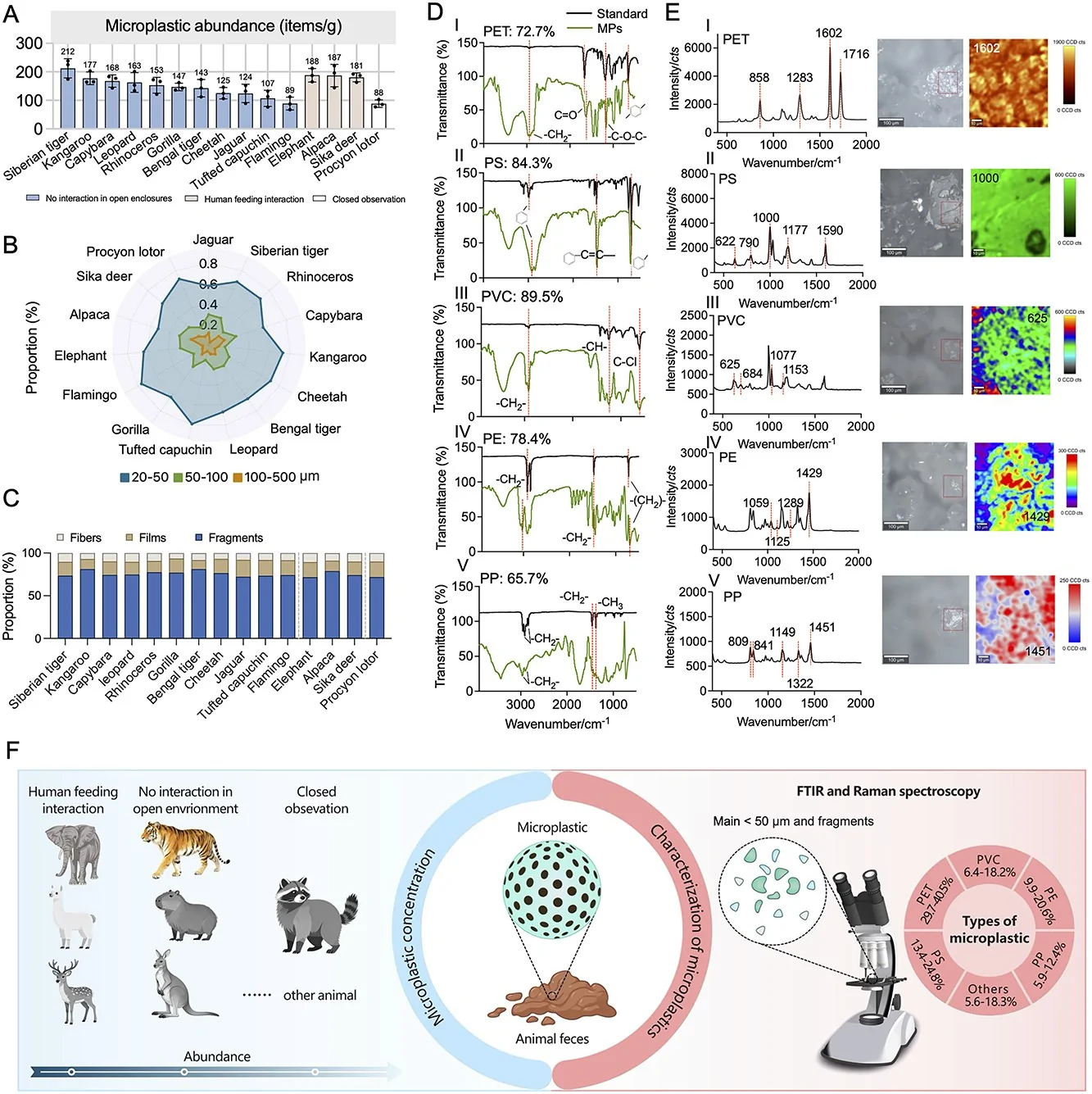
Characterization of MPs in animal feces. MP abundance (A), size distribution (B), and shape composition (C) in animal feces. (D) Representative FTIR spectra of MPs identified in this study. The upper trace represents the reference spectrum of the standard polymer material, whereas the lower trace represents the spectrum of the environmental MP sample. Polymer identification was performed using FTIR spectral matching against a reference library, and characteristic absorption peaks were used to confirm polymer types. (E) Optical image, representative Raman spectra, and Raman intensity maps of MP particles identified in this study. Raman spectra were collected using a 40× objective lens with an integration time of 1 s per pixel. Spectral mapping was performed with a spatial resolution of 0.33 × 0.33 μm, generating a 30 × 30 measurement matrix. Polymer types identified include PET, PS, PVC, PE, and PP. (F) Conceptual diagram summarizing the characterization of MPs in animal feces.

We surveyed feces from 15 representative zoo-dwelling terrestrial animal species to quantify intestinal MPs and assess their association with human–animal interaction regimes. Principal component analysis identified a clear correspondence between MP traits and three interaction categories (Fig. S3), defined by the degree and mode of visitor contact: human feeding interaction refers to enclosures where visitors were allowed to directly feed animals under zoo supervision; no interaction in open enclosures refers to open-display settings where visitors could observe animals but were not allowed to touch or feed them; and closed observation refers to barrier-separated enclosures where visitors could only observe animals through barriers, with no possibility of direct contact or feeding. MP abundance ranged from 88 to 212 items/g, with a predominant size of 20–50 μm (Fig. 1A and B). Siberian tiger, Elephant, and Alpaca had the highest MP loads, consistent with the intensive feeding interaction in these exhibits. Although MP size distributions did not differ significantly across species, comparisons based on the same or closely related taxa suggested that zoo animals tended to harbor higher fecal MP abundance than their wild counterparts (Table S1). For example, wild Amur tiger feces contained 21.37 items/g d.w [46]., whereas zoo Siberian tiger and Bengal tiger feces in our study contained 212 and 125 items/g d.w., respectively. Similarly, wild Sika deer and Roe deer contained 17.41 and 20.51 items/g d.w [46]., respectively, whereas zoo sika deer in our study contained 181 items/g d.w. For Elephant, a previous study reported 0.0182 items/g d.w. in wild *Elephas maximus* [47], whereas zoo Elephant in our study contained 188 items/g d.w. This divergence likely reflects elevated plastic exposure under captive feeding and management conditions and plastic ingestion and retention in animal digestive systems [48–50]. These findings indicate widespread ingestion and excretion of MPs and underscore the influence of human–animal interaction intensity on intestinal MP burdens. However, because published data permitting direct zoo–wild comparisons within the same species remain limited, this interpretation should be considered preliminary.

We next characterized MP morphology and polymer composition, given their importance for biofilm selection. Raman and FTIR analyses identified fragments as the dominant shape (>50%), followed by fibers and films (Fig. 1C). Polyethylene terephthalate (PET) and PS together comprised >65% of polymers, with PVC, PE, and PP also present (Fig. 1D–E and Fig. S4). This polymer profile is consistent with prior studies [51] and supports the notion that exhibit infrastructure, diet packaging, and visitor-related inputs influence MP exposure [52]. Because MP retention varies with diet, body size, and gut complexity [53–55], we selected feces from Siberian tiger (hereafter, “Tiger” is used throughout) and Elephant (highest MP concentrations) and the five most abundant polymer types detected in the fecal samples (PET, PS, PVC, PE, and PP) for intestinal simulation experiments (Fig. 1F).

### Microplastics select distinct, high-β-diversity plastisphere communities relative to gut microbiota

**Figure 2:**
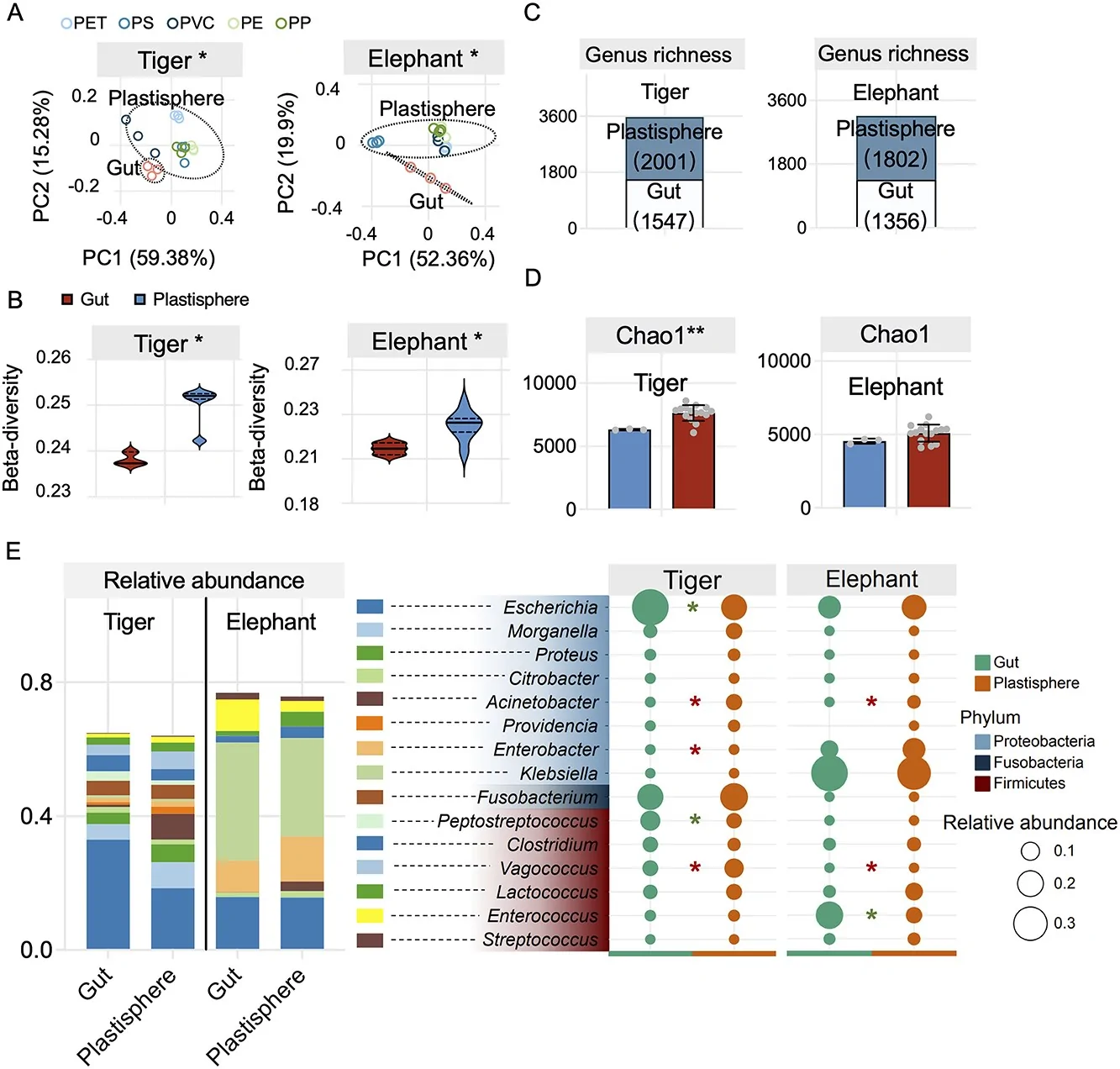
Distinct microbial communities in the plastisphere *vs.* animal gut. (A) Unconstrained PCoA of plastisphere and animal gut communities, with significance assessed using PERMANOVA (^*^P < .05, PERMANOVA). (B) Comparison of within-group beta-diversity, quantified as pairwise bray–Curtis dissimilarities among samples, between plastisphere and animal gut communities (^*^P < .05, Wilcoxon rank-sum test). Genus richness (C) and Chao1 diversity index (D) in Tiger and Elephant gut and plastisphere. (E) Stacked bar plots present the relative abundance of bacterial genera in gut and plastisphere samples. Bubble plots provide a more detailed comparison of genus-level relative abundance between gut and plastisphere in Tiger and Elephant samples (^*^P < .05, Wilcoxon rank-sum test).

To test whether MPs support microbial communities that differ from bulk gut microbiota, we established feces-derived intestinal simulations under anoxic conditions [28]. Scanning electron microscopy (SEM) confirmed dense biofilm formation on MP surfaces (Fig. S5). Although the washing step reduced loosely suspended cells, free-living microorganisms may not have been completely excluded. Therefore, the term “plastisphere” is used here in an operational sense to describe the microbial biofilm enriched on MP surfaces, consistent with SEM observations. Compared with the gut-inoculated treatments, MPs incubated without gut inoculum showed clean or only sparsely coated surfaces and lacked the dense biofilm observed in the experimental treatments. These results support our interpretation that the dense biofilms observed on MPs were mainly formed by gut-derived microorganisms. Metagenomic taxonomic profiling indicated that bacteria were the dominant microbial group in both gut and plastisphere fractions (Fig. S6). Unconstrained principal coordinate analysis (PCoA) demonstrated significant compositional separation between plastisphere and gut communities (Fig. 2A), although host-species effects (Tiger *vs.* Elephant) were stronger than the plastisphere-gut effect (Fig. S7). PS-associated plastispheres separated from the other polymers in both hosts, indicating a substrate effect on community assembly.

The plastisphere forms on organic, hydrophobic, buoyant, and persistent substrates that can supply nutrients to some microbes [56–58]. Under digestive conditions, however, MPs can release plastic-associated chemicals and facilitate the desorption of sorbed environmental contaminants, particularly hydrophobic organic pollutants such as polychlorinated biphenyls and polycyclic aromatic hydrocarbons, thereby imposing strong selection pressure [59–61]. Consequently, plastisphere biofilms selectively enrich microorganisms adapted to plastic-specific physiochemistry, creating distinct niches within the gut and promoting the formation of plastisphere ecosystems.

Plastisphere communities showed significantly higher *β*-diversity than gut communities (*P* < .05; Fig. 2B), indicating lower similarity among plastisphere samples. This greater community turnover is likely driven by plastic type [62, 63], physicochemical heterogeneity [57], limited dispersal, and substrate-driven selection, consistent with prior reports. Alpha-diversity analyses further demonstrated that plastisphere communities harbored higher richness and diversity than gut communities in both host species. In Tiger, genus richness was higher in plastisphere than in gut samples (2001 *vs.* 1547), whereas in Elephant the corresponding values were 1802 and 1356, respectively (Fig. 2C). The Chao1 index was higher in plastisphere than in gut communities for both Tiger and Elephant (Fig. 2D). These findings suggest that intestinal MPs can support richer and more compositionally diverse microbial assemblages than the surrounding gut environment.

At the genus level (Fig. 2E), Tiger gut communities were dominated by *Escherichia* (32.6%, average relative abundance), *Fusobacterium* (19.5%), and *Peptostreptococcus* (11.9%), whereas plastisphere communities showed increased relative abundances of *Acinetobacter*, *Enterobacter*, and *Vagococcus*, together with reduced relative abundances of *Escherichia* and *Peptostreptococcus*. Elephant gut communities were enriched in *Klebsiella* (34.2%), *Enterococcus* (22.3%), and *Escherichia* (19.2%), whereas *Acinetobacter* and *Enterobacter* were enriched on MPs. Given the clinical relevance of *Acinetobacter* and *Enterobacter* [64, 65], their enrichment on MPs suggests that intestinal MPs can act as selective carriers for opportunistic pathogens and potentially invasive taxa [16], thereby facilitating microbial dissemination from hosts to the environment. Together, these findings suggest that intestinal MPs may function as selective hotspots for microbial diversification and opportunistic pathogen enrichment, with potential implications for host-to-environment microbial transfer.

### ARGs, virulence factors, and pathogenic taxa are enriched in the plastisphere

**Figure 3:**
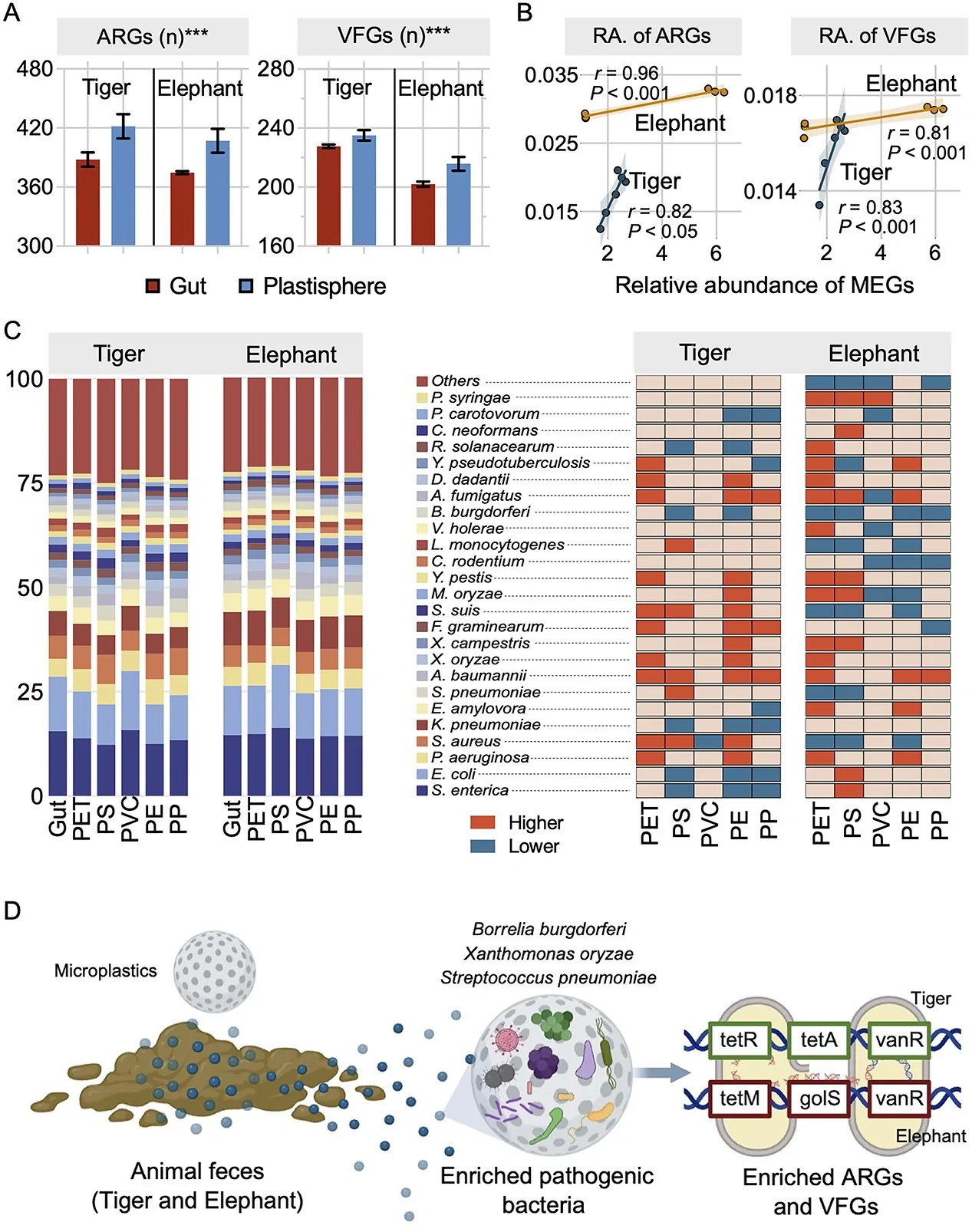
Quantification and comparative analysis of ARGs and VFGs in Tiger and Elephant gut *vs.* plastisphere. (A) Number of ARGs and VFGs detected in gut and plastisphere samples. (^***^P < .001, PERMANOVA). (B) Linear regression analysis between ARGs and VFGs abundances and MGEs in PS/PET-associated microbial communities. (C) Stacked bar plot of the relative abundance of potentially pathogenic bacterial species in gut and plastisphere samples. Only microbial species with >1% abundance and significant differences (*P* < .05, one-way ANOVA) are presented. In the right-hand comparison grid, cells categorized as Lower or Higher indicate species abundance significantly lower or higher than the corresponding control, respectively (*P* < .05). (D) Conceptual diagram illustrating the potential enrichment and transfer patterns of plastisphere-associated microorganisms in animal gut environments. Created in BioRender. Luo, Z. (2026). https://BioRender.com/72n9nfu

Metagenomic profiling revealed a pronounced enrichment of ARGs and VFGs in plastisphere communities relative to gut microbiota in both Tiger and Elephant (*P* < .05; Fig. 3A). Across five polymers, we detected 465 ARG subtypes conferring resistance to nine antibiotic classes. Overall, the class-level ARG distribution patterns were broadly consistent in both host species and across all polymer types, with no notable exceptions observed. Tetracycline-, fluoroquinolone-, and macrolide-related ARGs were most prevalent, with tetracycline ARGs significantly higher in gut-derived plastispheres (Fig. S8). In Tiger, *tetR*, *tetA* (tetracycline), and *vanR* (glycopeptide) were 3.0–8.7 times higher in plastispheres than in gut, and fluoroquinolone resistance genes were enriched across all polymers. In Elephant, *tetM* (tetracycline), *vanR* (glycopeptide), and *golS* (penam) increased 6.0–10.9-fold in plastispheres (Fig. S9). Mechanistically, enriched ARGs were dominated by efflux and inactivation pathways, consistent with multidrug resistance strategies.

VFG abundances were likewise elevated in plastispheres (*P* < .05; Fig. 3A). Total ARG and VFG abundance was strongly and positively correlated with MGEs on PET/PS (*r* > 0.80, *P* < .05, Fig. 3B), indicating enhanced horizontal gene transfer potential in MP biofilms. At the species level, plastispheres harbored significantly higher counts of potentially pathogenic species than gut microbiota (*P* < .05; Figs. 3C and S10), with polymer-specific patterns. In Tiger samples, PET, PS, and PE were associated with higher abundances of *Borrelia burgdorferi, Xanthomonas oryzae,* and *Streptococcus pneumoniae* relative to gut (*P* < .05); PVC and PP showed weaker effects. Similar trends were observed in Elephant. Collectively, these results demonstrate that intestinal MPs act as selective hotspots for potential pathogens, ARGs, and VFGs [66–68], increasing the likelihood of environmental dissemination (Fig. 3D).

### Secretion systems and EPS underpin enhanced pathogenic potential on MPs

**Figure 4:**
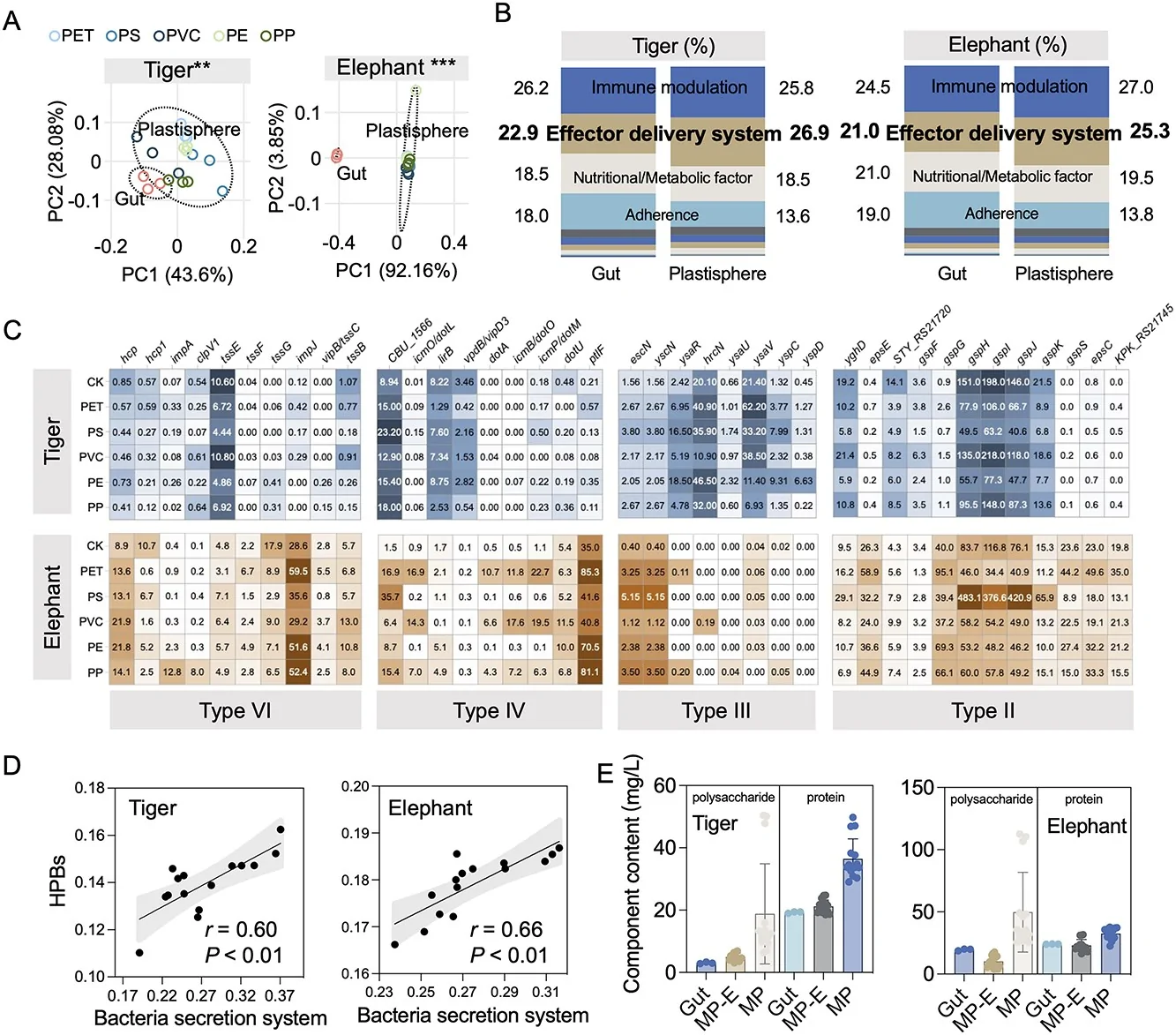
Differentiation of bacterial secretion systems in plastisphere *vs.* animal gut. (A) Unconstrained PCoA of bacterial secretion system gene profiles, with statistical significance assessed using PERMANOVA (^**^P < .01, ^***^P < .001), indicating clear separation between plastisphere and animal gut samples. (B) Stacked bar plot showing the proportion of total VFGs classified by the function and mechanism in Tiger and Elephant gut and plastisphere. (C) Heat map showing the relative abundance of bacterial secretion system (Types II, III, IV, and VI) genes in Tiger and Elephant gut and plastisphere. Gene abundances are presented as original abundance × 10^3^ for comparative visualization. (D) Linear-regression analysis between secretion system gene abundance and host-associated pathogenic bacteria (HPBs) in the plastisphere samples. (E) Composition of EPS in Tiger and Elephant gut and plastisphere samples, based on biochemical quantification of major macromolecular components.

To elucidate mechanisms of pathogenicity in plastisphere biofilms, we profiled secretion systems and VFG categories, supported by network and metabolic annotations. In gut communities, VFGs were dominated by immune modulation (Tiger 26.2%, Elephant 24.5%; Fig. 4B). In the plastisphere, effector delivery-related VFGs rose to 26.9% in Tiger and 25.3% in Elephant, mirroring significant enrichment of Type II, III, IV, and VI secretion system genes across polymers (Fig. 4C). Co-occurrence networks identified stress survival, regulation, and adherence as key nodes in Tiger gut, with plastisphere networks adding biofilm formation and effector delivery as central hubs (Fig. S11); similar patterns were observed in Elephant (Fig. S12). Plastispheres in both Tiger and Elephant were highly enriched in secretion system genes (Fig. 4C). Kyoto Encyclopedia of Genes and Genomes (KEGG)-based functional annotation corroborated the enhanced secretion system potential of plastispheres (Fig. S13). Unconstrained PCoA demonstrated that plastisphere gene profiles differed significantly from gut communities (Fig. 4A), and niche breadth analyses indicated broader niches in both hosts (Fig. S14), offering greater opportunity for secretion system assembly and biofilm development.

Mechanistically, Type II, III, and VI systems deliver effectors that promote colonization and invasion [69] (e.g. *Vibrio cholerae* and enterotoxigenic and enterohemorrhagic *Escherichia coli* [70–72]), whereas Type IV mediates conjugation, DNA exchange, and effector secretion, enhancing adaptability [73]. Multiple secretion systems are used by pathogens, including *Vibrio parahaemolyticus* [74] and *Pseudomonas aeruginosa* [75], which use both Type III and Type VI systems. Secretion system gene abundance on MPs correlated strongly with human pathogenic bacteria (Fig. 4D), implicating secretion machinery in pathogen enrichment. Additionally, EPS compound concentrations were higher in the plastisphere than in gut communities (Tiger: polysaccharides +567.1% *vs* gut and 379.3% *vs* environment; proteins +188.8% and 171.9%; Elephant: polysaccharides +254.3% and + 489.4%; proteins +134.7% and + 141.2%; Fig. 4E), consistent with previous studies [76, 77] and indicating that EPS-rich matrices retain ARGs, promote cell cohesion, and foster horizontal ARG transfer. Collectively, plastisphere biofilms couple enriched secretion systems that drive pathogenicity with abundant EPS that stabilizes communities and facilitates gene exchange, enhancing pathogen persistence and dissemination on MP surfaces.

### Gut-derived plastispheres show prolonged environmental survival and functional shifts

**Figure 5:**
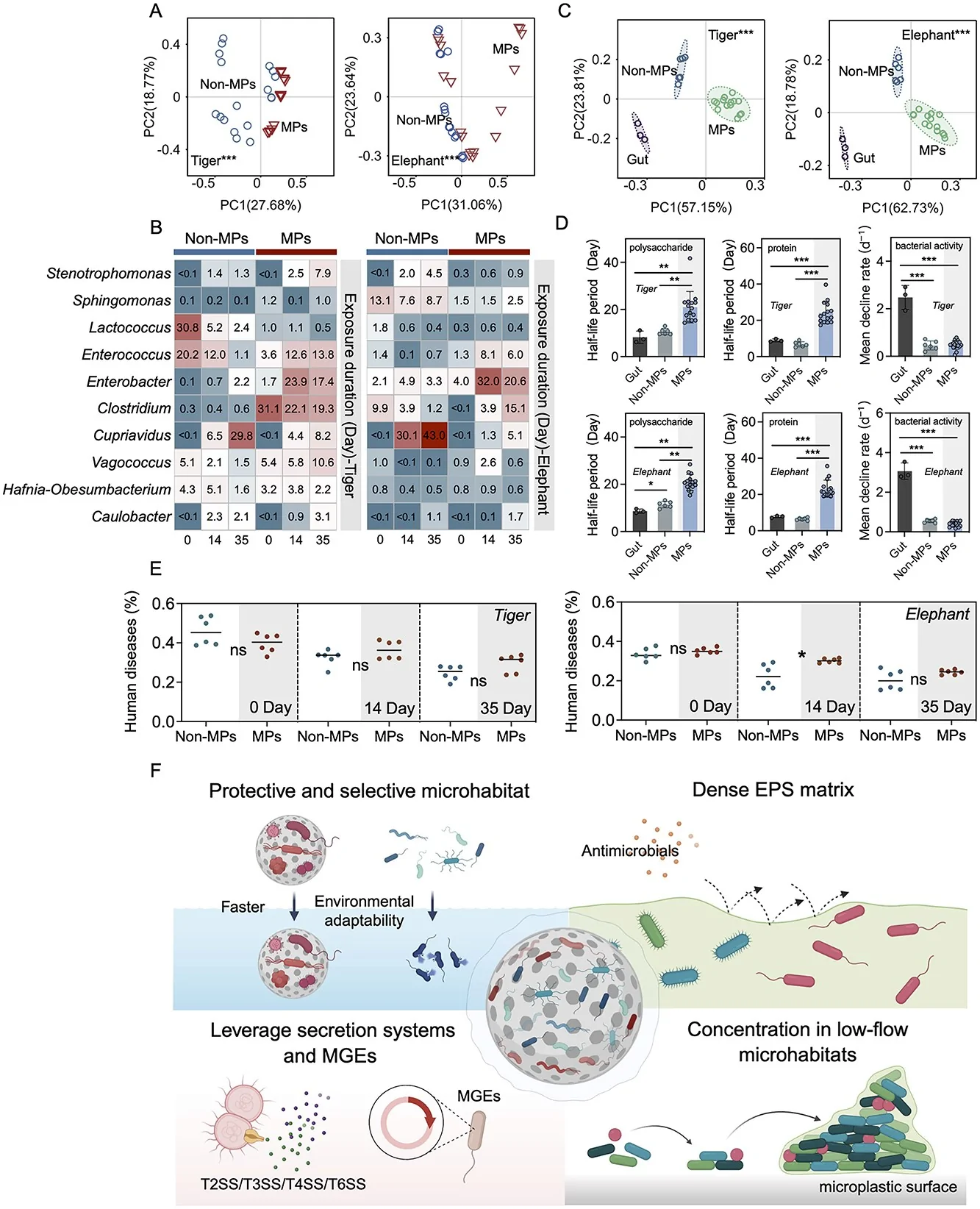
Microbial dynamics and potential pathogen enrichment on plastispheres in animal feces during environmental exposure based on 16S rRNA gene sequencing. (A) Unconstrained PCoA showing that the MPs harbored bacterial communities distinct from those on the non-MPs (^***^P < .001, PERMANOVA). (B) Heat map of abundant potentially pathogenic bacteria in MP and non-MP samples from Tiger and Elephant intestinal simulation systems at 0, 14, and 35 days. Data are presented as relative abundance (%). (C) Unconstrained PCoA of temporal variation in biofilm protein, polysaccharide of biofilms, and bacterial activity profiles from MPs, non-MPs, and gut bacteria during 0–35 days (^***^P < .001, PERMANOVA). (D) Half-life period of polysaccharides and proteins and average decline rates of bacterial activity (× 10^3^) in MPs, non-MPs, and gut bacteria from day 0 to day 35. (E) Temporal changes in KEGG level 1 pathways assigned to human diseases in MPs and non-MPs from Tiger and Elephant during environmental exposure. (F) Conceptual model illustrating the multiple changes in the animal feces plastisphere microbiome from Tiger and Elephant intestinal simulation systems. Created in BioRender. Luo, Z. (2026). https://BioRender.com/2wnqest

To assess the environmental persistence of gut-derived MP-associated communities, we compared them with communities associated with non-plastic microparticles (non-MPs; biochar and SiO₂) and with free gut microbiota during 35 days of water exposure. Across the exposure period, MP-associated communities consistently exhibited greater persistence than both non-MP-associated communities and free gut microbiota (Fig. S15), indicating that plastic surfaces provided a more protective habitat for attached bacteria [6, 8, 78]. PET and PS showed the two lowest mean bacterial activity decline rates over the 35-day exposure period (PET: 0.397 × 10^3^ day^−1^; PS: 0.440 × 10^3^ day^−1^; Fig. S15) among the tested polymers, indicating greater persistence than the other polymers and justifying their selection for subsequent community profiling. Community succession also differed between particle types. The Chao1 index declined continuously on non-MPs, suggesting a progressive loss of taxa during exposure, whereas richness on MPs increased at day 14 and then decreased by day 35, indicating a transient expansion of colonizers followed by later-stage selection [79, 80] (Fig. S17). Consistently, PCoA revealed clear separation between MP- and non-MP-associated communities in both Tiger and Elephant samples (Fig. 5A), demonstrating that plastic and non-plastic particles selected for distinct microbial assemblages during environmental exposure. This difference was further reflected in the dynamics of potentially pathogenic taxa: *Enterococcus* (includes vancomycin resistance genes [81]) and *Clostridium* decreased over time on non-MPs but increased on MPs, whereas *Enterobacter* showed only a slight increase on non-MPs but was enriched on MPs (Fig. 5B). Together, these results indicate that MPs not only served as colonization surfaces but also imposed a stronger ecological selection that favored the persistence and enrichment of potentially pathogenic bacteria.

Biofilm traits and functional predictions further supported MP-specific persistence. Protein content, polysaccharide content, and bacterial activity all changed significantly over time and followed distinct trajectories among gut bacteria, non-MPs, and MPs (Fig. 5C and D; Figs. S15 and S16). The half-lives of proteins and polysaccharides were substantially longer on MPs than on non-MPs or in free gut communities, indicating that plastic surfaces maintained a more stable EPS matrix [82]. Although bacterial activity declined in all treatments, the decrease was much slower in particle-associated communities than in free gut microbiota and was generally slower on MPs than on non-MPs (Fig. S15). KEGG functional prediction showed that the pathway relative to human diseases and antibiotic biosynthesis declined over time in both MP and non-MP treatments but decreased more slowly on MPs, including pathways associated with ansamycin and vancomycin-group biosynthesis (Figs. 5E and S18). This pattern suggests prolonged retention of disease-related functional potential on plastic surfaces. Collectively, these findings show that the stronger persistence of potentially pathogenic taxa on MPs cannot be attributed solely to generic biofilm formation on suspended particles. The specific physicochemical properties of MPs likely create a more favorable and selective habitat for gut-derived biofilms, thereby enhancing the environmental persistence and potential dissemination of opportunistic pathogens and resistance-related traits. Conceptually (Fig. 5F), gut-derived plastispheres may (i) stabilize and protect pathogens against environmental stressors (e.g. UV, temperature) [83, 84], (ii) maintain dense EPS matrices that hinder antimicrobial penetration and control [52], (iii) concentrate pathogens and genes in low-flow microhabitats [7], and (iv) leverage secretion systems and MGEs to persist and potentially spread resistance. Given that zoo feces can enter adjacent water bodies, these persistent, functionally fortified plastispheres elevate the risk of environmental and waterborne transmission to other animals and humans.

## Conclusion

The findings show how MPs shape intestinal microbiomes in zoo-dwelling higher-terrestrial megafauna and clarify their ecological and public health implications. We show that MP characteristics track human–animal interaction patterns, with zoo feces harboring more abundant and diverse MPs than those of wild counterparts. Within the gut, MPs foster unique plastisphere communities that differ from bulk microbiota, elevate risks of invasive species transfer, and disrupt ecosystem stability. In both Tiger and Elephant, plastispheres were enriched in ARGs, pathogenic taxa, virulence factors, and secretion system genes, collectively amplifying pathogenic potential and facilitating ARG retention. Genera such as *Enterococcus*, *Enterobacter*, and *Clostridium* exhibited prolonged survivability and heightened predicted virulence potential in ambient aquatic settings, raising concerns about downstream waterborne infections. The observed effects cannot be attributed solely to generic biofilm formation because plastic surfaces selected for distinct and more persistent microbial colonizers than non-plastic particles. These findings call for deeper investigation into MP-microbe interactions, including longitudinal studies to assess the long-term effects on wildlife health and ecosystem dynamics. They also highlight the need for coordinated strategies to mitigate MP pollution and implement evidence-based management practices, achieved through collaboration among researchers, policymakers, and conservationists to safeguard environmental and public health.

## Supplementary Material

Web_Material_wrag207

## Data Availability

The raw metagenomic sequence data have been deposited in the NCBI Sequence Read Archive under BioProject accession PRJNA1413497. The raw 16S rRNA gene amplicon sequence data have been deposited under BioProject accession PRJNA1475087. Both datasets are publicly available. All additional data supporting the findings of this study are provided in the article and its Supplementary Material file.

## References

[1] Bank MS, Hansson SV. The plastic cycle: a novel and holistic paradigm for the Anthropocene. *Environ Sci Technol* 2019;53:7177–9. 10.1021/acs.est.9b02942 31198029

[2] Sun XD, Yuan XZ, Jia Y, et al. Differentially charged nanoplastics demonstrate distinct accumulation in *Arabidopsis thaliana*. *Nat Nanotechnol* 2020;15:755–60. 10.1038/s41565-020-0707-4 32572228

[3] Luo Y, Li L, Feng Y, et al. Quantitative tracing of uptake and transport of submicrometre plastics in crop plants using lanthanide chelates as a dual-functional tracer. *Nat Nanotechnol* 2022;17:424–31. 10.1038/s41565-021-01063-3 35058654

[4] D’Souza JM, Windsor FM, Santillo D, et al. Food web transfer of plastics to an apex riverine predator. *Glob Change Biol* 2020;26:3846–57. 10.1111/gcb.15139 32441452

[5] Yan Z, Liu Y, Zhang T, et al. Analysis of microplastics in human feces reveals a correlation between fecal microplastics and inflammatory bowel disease status. *Environ Sci Technol* 2022;56:414–21. 10.1021/acs.est.1c03924 34935363

[6] Amaral-Zettler LA, Zettler ER, Mincer TJ. Ecology of the plastisphere. *Nat Rev Microbiol* 2020;18:139–51. 10.1038/s41579-019-0308-0 31937947

[7] Zettler ER, Mincer TJ, Amaral-Zettler LA. Life in the “Plastisphere”: microbial communities on plastic marine debris. *Environ Sci Technol* 2013;47:7137–46. 10.1021/es401288x 23745679

[8] Sun Y, Wu M, Xie S, et al. Homogenization of bacterial plastisphere community in soil: a continental-scale microcosm study. *ISME Commun* 2024;4:ycad012. 10.1093/ismeco/ycad012 PMC10848224 38328447

[9] Lo LSH, Liu X, Qian P-Y, et al. Microbial colonization and chemically influenced selective enrichment of bacterial pathogens on polycarbonate plastic. *Environ Sci Pollut Res* 2024;31:8061–71. 10.1007/s11356-023-31752-6 38175506

[10] Witsø IL, Basson A, Vinje H, et al. Freshwater plastispheres as a vector for foodborne bacteria and viruses. *Environ Microbiol* 2023;25:2864–81. 10.1111/1462-2920.16536 37964725

[11] Wang YF, Liu YJ, Fu YM, et al. Microplastic diversity increases the abundance of antibiotic resistance genes in soil. *Nat Commun* 2024;15:9788. 10.1038/s41467-024-54237-7 PMC11557862 39532872

[12] Lin D, Xu JY, Wang L, et al. Long-term application of organic fertilizer prompting the dispersal of antibiotic resistance genes and their health risks in the soil plastisphere. *Environ Int* 2024;183:108431. 10.1016/j.envint.2024.108431 38217904

[13] Li C, Liu J, Rillig MC, et al. What harmful microbes are lurking in the world’s 7 billion tonnes of plastic waste? *Nature* 2024;634:30–2. 10.1038/d41586-024-03150-6 39354074

[14] Ni B, Zhang TL, Cai TG, et al. Effects of heavy metal and disinfectant on antibiotic resistance genes and virulence factor genes in the plastisphere from diverse soil ecosystems. *J Hazard Mater* 2024;465:133335. 10.1016/j.jhazmat.2023.133335 38142651

[15] Li C, Li X., Bank, M. S, et al. The “microplastome”–a holistic perspective to capture the real-world ecology of microplastics. *Environ Sci Technol* 2024;58:4060–9. 10.1021/acs.est.3c08849 PMC10919093 38331396

[16] Li C, Gillings MR, Zhang C, et al. Ecology and risks of the global plastisphere as a newly expanding microbial habitat. *Innovation* 2024;5:100543. 10.1016/j.xinn.2023.100543 PMC10726253 38111463

[17] Zhu D, Ma J, Li G, et al. Soil plastispheres as hotspots of antibiotic resistance genes and potential pathogens. *ISME J* 2022;16:521–32. 10.1038/s41396-021-01103-9 PMC8776808 34455424

[18] Luo T, Dai X, Wei W, et al. Microplastics enhance the prevalence of antibiotic resistance genes in anaerobic sludge digestion by enriching antibiotic-resistant bacteria in surface biofilm and facilitating the vertical and horizontal gene transfer. *Environ Sci Technol* 2023;57:14611–21. 10.1021/acs.est.3c02815 37733635

[19] Kirstein IV, Kirmizi S, Wichels A, et al. Dangerous hitchhikers? Evidence for potentially pathogenic *vibrio* spp. on microplastic particles. *Mar Environ Res* 2016;120:1–8. 10.1016/j.marenvres.2016.07.004 27411093

[20] Wu X, Pan J, Li M, et al. Selective enrichment of bacterial pathogens by microplastic biofilm. *Water Res* 2019;165:114979. 10.1016/j.watres.2019.114979 31445309

[21] Avio CG, Gorbi S, Regoli F. Experimental development of a new protocol for extraction and characterization of microplastics in fish tissues: first observations in commercial species from the Adriatic Sea. *Mar Environ Res* 2015;111:18–26. 10.1016/j.marenvres.2015.06.014 26210759

[22] Bessa F, Ratcliffe N, Otero V, et al. Microplastics in gentoo penguins from the Antarctic region. *Sci Rep* 2019;9:14191. 10.1038/s41598-019-50621-2 PMC6775258 31578393

[23] Bessa F, Frias JPGL, Kögel T, et al. Harmonized protocol for monitoring microplastics in biota. *JPI-Oceans BASEMAN project* 2019; Deliverable 4.3,30.

[24] Le Guen C, Suaria G, Sherley RB, et al. Microplastic study reveals the presence of natural and synthetic fibres in the diet of king penguins (*Aptenodytes patagonicus*) foraging from South Georgia. *Environ Int* 2020;134:105303. 10.1016/j.envint.2019.105303 31726359

[25] AOAC International . Official method 920.39. Fat (crude) or ether extract in animal feed. In: Latimer GW, Jr. (ed.), Official Methods of Analysis of AOAC International. 21st edn. ( AOAC International, Rockville, MD, 2019), 10.1007/s40201-019-00403-9.

[26] AMF , Materić D, Valsami-Jones E, et al. Challenges in studying microplastics in human brain. *Nat Med* 2025;31:4034–5. 10.1038/s41591-025-04045-3 41233597

[27] Lei J, Zhang X, Yan W, et al. Urban microplastic pollution revealed by a large-scale wetland soil survey. *Environ Sci Technol* 2023;57:8035–43. 10.1021/acs.est.2c08567 37200099

[28] Van den Abbeele P, Grootaert C, Marzorati M, et al. Microbial community development in a dynamic gut model is reproducible, colon region specific, and selective for *Bacteroidetes* and *clostridium* cluster IX. *Appl Environ Microbiol* 2010;76:5237–46. 10.1128/AEM.00759-10 PMC2916472 20562281

[29] Grootaert C, Van den Abbeele P, Marzorati M, et al. Comparison of prebiotic effects of arabinoxylan oligosaccharides and inulin in a simulator of the human intestinal microbial ecosystem. *FEMS Microbiol Ecol* 2009;69:231–42. 10.1111/j.1574-6941.2009.00712.x 19508502

[30] Possemiers S, Rabot S, Espín JC, et al. *Eubacterium limosum* activates isoxanthohumol from hops (*Humulus lupulus* L.) into the potent phytoestrogen 8-prenylnaringenin in vitro and in rat intestine. *J Nutr* 2008;138:1310–6. 10.1093/jn/138.7.1310 18567753

[31] Wang D, Jiang Y, Wu D, et al. Health effect of N-nitroso diethylamine in treated water on gut microbiota using a simulated human intestinal microbiota system. *Processes* 2022;10:438. 10.3390/pr10030438

[32] Molly K, Vande Woestyne M, Verstraete W. Development of a 5-step multi-chamber reactor as a simulation of the human intestinal microbial ecosystem. *Appl Microbiol Biotechnol* 1993;39:254–8. 10.1007/BF00228615 7763732

[33] Possemiers S, Verthé K, Uyttendaele S, et al. PCR-DGGE-based quantification of stability of the microbial community in a simulator of the human intestinal microbial ecosystem. *FEMS Microbiol Ecol* 2004;49:495–507. 10.1016/j.femsec.2004.05.002 19712298

[34] Teng Z, Shao W, Zhang K, et al. Pb biosorption by *Leclercia adecarboxylata*: protective and immobilized mechanisms of extracellular polymeric substances. *Chem Eng J* 2019;375:122113. 10.1016/j.cej.2019.122113

[35] Raunkjær K, Hvitved-Jacobsen T, Nielsen PH. Measurement of pools of protein, carbohydrate and lipid in domestic wastewater. *Water Res* 1994;28:251–62. 10.1016/0043-1354(94)90261-5

[36] Bradford MM . A rapid and sensitive method for the quantitation of microgram quantities of protein utilizing the principle of protein-dye binding. *Anal Biochem* 1976;72:248–54. 10.1016/0003-2697(76)90527-3 942051

[37] Chen S, Zhou Y, Chen Y, et al. Fastp: an ultra-fast all-in-one FASTQ preprocessor. *Bioinformatics* 2018;34:i884–90. 10.1093/bioinformatics/bty560 PMC6129281 30423086

[38] Hyatt D, Chen G-L, LoCascio PF, et al. Prodigal: prokaryotic gene recognition and translation initiation site identification. *BMC Bioinformatics* 2010;11:119. 10.1186/1471-2105-11-119 PMC2848648 20211023

[39] Huang Q, Wu H, Cai P, et al. Atomic force microscopy measurements of bacterial adhesion and biofilm formation onto clay-sized particles. *Sci Rep* 2015;5:16857. 10.1038/srep16857 PMC4653644 26585552

[40] Flemming H-C, Wuertz S. Bacteria and archaea on earth and their abundance in biofilms. *Nat. Rev. Microbiol.* 2019;17:247–60. 10.1038/s41579-019-0158-9 30760902

[41] Thies JE, Rillig MC. Characteristics of biochar: Biological properties. In: Lehmann J, Joseph S. (eds.), Biochar for Environmental Management 85–105. (Earthscan, London) 2009.

[42] Lehmann J, Rillig MC, Thies J, et al. Biochar effects on soil biota–a review. *Soil Biol Biochem* 2011;43:1812–36. 10.1016/j.soilbio.2011.04.022

[43] Wang G, Cui C, Wang Y, et al. Enhancement of the anaerobic biodegradation efficiency of azo dye by anthraquinone-loaded biochar biofilm: factors affecting biofilm formation and the enhancement mechanism. *Biochar* 2024;6:95.

[44] Cong S, Tong Q, Peng Q, et al. *In vitro* anti-bacterial activity of diosgenin on Porphyromonas gingivalis and Prevotella intermedia. *Mol Med Rep* 2020;22:5392–8. 10.3892/mmr.2020.11620 PMC7647021 33174005

[45] Langille MGI, Zaneveld J, Caporaso JG, et al. Predictive functional profiling of microbial communities using 16S rRNA marker gene sequences. *Nat Biotechnol* 2013;31:814–21. 10.1038/nbt.2676 PMC3819121 23975157

[46] Huang Z, Liu D, Cheng W, et al. Microplastics in the Amur tiger's habitat: occurrence, characteristics, and risk assessment. *J Hazard Mater* 2025;493:138380. 10.1016/j.jhazmat.2025.138380 40288321

[47] Teampanpong J, Duengkae P. Using feces to indicate plastic pollution in terrestrial vertebrate species in western Thailand. *PeerJ* 2024;12:e17596. 10.7717/peerj.17596 PMC11212639 38948236

[48] Browne MA, Dissanayake A, Galloway TS, et al. Ingested microscopic plastic translocates to the circulatory system of the mussel, *Mytilus edulis* (L.). *Environ Sci Technol* 2008;42:5026–31. 10.1021/es800249a 18678044

[49] Bayhan B, Uncumusaoglu AA. Abundance, characteristics and potential ecological risks of microplastics in some commercial fish in İzmir Bay (Aegean Sea, Türkiye). *Reg Stud Mar Sci* 2024;73:103488.

[50] Peng X, Yang T, Guo S, et al. Revealing chemical release from plastic debris in animals’ digestive systems using nontarget and suspect screening and simulating digestive fluids. *Environ Pollut* 2024;348:123793. 10.1016/j.envpol.2024.123793 38513944

[51] Pérez-Flores J, Borges-Ramírez MM, Vargas-Contreras JA, et al. Inter-annual variation in the microplastics abundance in feces of the Baird’s tapir (*Tapirus bairdii*) from the Selva Maya. *México Sci Total Environ* 2024;941:173659. 10.1016/j.scitotenv.2024.173659 38839015

[52] Guo Y, Liu Y, Xiang T, et al. Disposable polypropylene face masks: a potential source of micro/nanoparticles and organic contaminates in humans. *Environ Sci Technol* 2023;57:5739–50. 10.1021/acs.est.2c06802 36989422

[53] Porter A, Godbold JA, Lewis CN, et al. Microplastic burden in marine benthic invertebrates depends on species traits and feeding ecology within biogeographical provinces. *Nat Commun* 2023;14:8023. 10.1038/s41467-023-43788-w PMC10696022 38049431

[54] Watts AJR, Lewis C, Goodhead RM, et al. Uptake and retention of microplastics by the shore crab *Carcinus maenas*. *Environ Sci Technol* 2014;48:8823–30. 10.1021/es501090e 24972075

[55] Joyce H, Frias J, Kavanagh F, et al. Plastics, prawns, and patterns: microplastic loadings in *Nephrops norvegicus* and surrounding habitat in the north East Atlantic. *Sci Total Environ* 2022;826:154036. 10.1016/j.scitotenv.2022.154036 35202687

[56] Ning D, Deng Y, Tiedje JM, et al. A general framework for quantitatively assessing ecological stochasticity. *Proc Natl Acad Sci USA* 2019;116:16892–8. 10.1073/pnas.1904623116 PMC6708315 31391302

[57] Louca S, Parfrey LW, Doebeli M. Decoupling function and taxonomy in the global ocean microbiome. *Science* 2016;353:1272–7. 10.1126/science.aaf4507 27634532

[58] Zhang X, Li Y, Ouyang D, et al. Systematical review of interactions between microplastics and microorganisms in the soil environment. *J Hazard Mater* 2021;418:126288. 10.1016/j.jhazmat.2021.126288 34102358

[59] Baj J, Dring JC, Czeczelewski M, et al. Derivatives of plastics as potential carcinogenic factors: the current state of knowledge. *Cancers* 2022;14:4637. 10.3390/cancers14194637 PMC9562888 36230560

[60] Mohamed Nor NH, Niu Z, Hennebelle M, et al. How digestive processes can affect the bioavailability of PCBs associated with microplastics: a modelling study supported by empirical data. *Environ Sci Technol* 2023;57:11452–64. 10.1021/acs.est.3c02129 PMC10413949 37504896

[61] Mohamed Nor NH, Koelmans AA. Transfer of PCBs from microplastics under simulated gut fluid conditions is biphasic and reversible. *Environ Sci Technol* 2019;53:1874–83. 10.1021/acs.est.8b05143 30638363

[62] Zhang J, Liu YX, Zhang N, et al. *NRT1.1B* is associated with root microbiota composition and nitrogen use in field-grown rice. *Nat Biotechnol* 2019;37:676–84. 10.1038/s41587-019-0104-4 31036930

[63] Yuan J, Wen T, Zhang H, et al. Predicting disease occurrence with high accuracy based on soil macroecological patterns of Fusarium wilt. *ISME J* 2020;14:2936–50. 10.1038/s41396-020-0720-5 PMC7784920 32681158

[64] Ahmadinejad M, Mohammadzadeh S, Pak H, et al. Bronchoalveolar lavage of ventilator-associated pneumonia patients for antibiotic resistance and susceptibility test. *Health Sci Rep* 2022;5:e472. 10.1002/hsr2.472 PMC8733848 35024459

[65] Umesha S, Manukumar HM. Advanced molecular diagnostic techniques for detection of food-borne pathogens: current applications and future challenges. *Crit Rev Food Sci Nutr* 2018;58:84–104. 10.1080/10408398.2015.1126701 26745757

[66] Prestholdt TE, Kemp L. The effects of anthropogenic marine debris on the behavior of the purple shore crab *Hemigrapsus nudus*. *J Sea Res* 2020;163:101916. 10.1016/j.seares.2020.101916

[67] Choi H, Im DH, Park YH, et al. Ingestion and egestion of polystyrene microplastic fragments by the Pacific oyster Crassostrea gigas. *Environ Pollut* 2022;307:119217. 10.1016/j.envpol.2022.119217 35421553

[68] Wang J, Lu L, Sun Y, et al. AHL-mediated quorum sensing drives plastisphere formation and elevates pathogenic potential. *ISME J.* 2026;20:wrag066. 10.1093/ismejo/wrag066 PMC13122622 41874421

[69] Galán JE, Wolf-Watz H. Protein delivery into eukaryotic cells by type III secretion machines. *Nature* 2006;444:567–73. 10.1038/nature05272 17136086

[70] Nivaskumar M, Francetic O. Type II secretion system: a magic beanstalk or a protein escalator. *Biochim Biophys Acta* 2014;1843:1568–77. 10.1016/j.bbamcr.2013.12.020 24389250

[71] Kulkarni R, Dhakal BK, Slechta ES, et al. Roles of putative type II secretion and type IV pilus systems in the virulence of uropathogenic *Escherichia coli*. *PLoS One* 2009;4:e4752. 10.1371/journal.pone.0004752 PMC2649431 19270734

[72] Lathem WW, Grys TE, Witowski SE, et al. StcE, a metalloprotease secreted by *Escherichia coli* O157:H7, specifically cleaves C1 esterase inhibitor. *Mol Microbiol* 2002;45:277–88. 10.1046/j.1365-2958.2002.02997.x 12123444

[73] Meng R, Jiang M, Cui Z, et al. Structural basis for the adsorption of a single-stranded RNA bacteriophage. *Nat Commun* 2019;10:3130. 10.1038/s41467-019-11126-8 PMC6635492 31311931

[74] Li P, Kinch LN, Ray A, et al. Acute hepatopancreatic necrosis disease-causing *Vibrio parahaemolyticus* strains maintain an antibacterial type VI secretion system with versatile effector repertoires. *Appl Environ Microbiol* 2017;83:e00737–17. 10.1128/AEM.00737-17 PMC5478980 28432099

[75] Moscoso JA, Mikkelsen H, Heeb S, et al. The Pseudomonas aeruginosa sensor RetS switches type III and type VI secretion via c-di-GMP signalling. *Environ Microbiol* 2011;13:3128–38. 10.1111/j.1462-2920.2011.02595.x 21955777

[76] Shiu RF, Vazquez CI, Chiang CY, et al. Nano- and microplastics trigger secretion of protein-rich extracellular polymeric substances from phytoplankton. *Sci Total Environ* 2020;748:141469. 10.1016/j.scitotenv.2020.141469 33113698

[77] Hung CM, Chen CW, Huang CP, et al. Ecological responses of coral reef to polyethylene microplastics in community structure and extracellular polymeric substances. *Environ Pollut* 2022;307:119522. 10.1016/j.envpol.2022.119522 35640726

[78] Oberbeckmann S, Osborn AM, Duhaime MB. Microbes on a bottle: substrate, season and geography influence community composition of microbes colonizing marine plastic debris. *PLoS One* 2016;11:e0159289. 10.1371/journal.pone.0159289 PMC4972250 27487037

[79] Battin TJ, Besemer K, Bengtsson MM, et al. The ecology and biogeochemistry of stream biofilms. *Nat. Rev. Microbiol.* 2016;14:251–63. 10.1038/nrmicro.2016.15 26972916

[80] Dang H, Lovell CR. Microbial surface colonization and biofilm development in marine environments. *Microbiol Mol Biol Rev* 2016;80:91–138. 10.1128/MMBR.00037-15 PMC4711185 26700108

[81] Arias CA, Murray BE. The rise of the enterococcus: beyond vancomycin resistance. *Nat Rev Microbiol* 2012;10:266–78. 10.1038/nrmicro2761 PMC3621121 22421879

[82] Arias-Andres M, Kettner MT, Miki T, et al. Microplastics: new substrates for heterotrophic activity contribute to altering organic matter cycles in aquatic ecosystems. *Sci Total Environ* 2018;635:1152–9. 10.1016/j.scitotenv.2018.04.199 29710570

[83] Sathicq MB, Sabatino R, Corno G, et al. Are microplastic particles a hotspot for the spread and the persistence of antibiotic resistance in aquatic systems? *Environ Pollut* 2021;279:116896. 10.1016/j.envpol.2021.116896 33744628

[84] Lehel J, Murphy S. Microplastics in the food chain: food safety and environmental aspects. *Rev Environ Contam Toxicol* 2021;259:1–49. 10.1007/398_2021_77 34611754

