# Supplementary material for: Zoo gut plastispheres enable pathogen escape and adaptation
Source: ISME J. 2026 Aug 5;20(1):wrag207. doi: 10.1093/ismejo/wrag207 (PMC13590889; doi:10.1093/ismejo/wrag207)
Supplement: Supplementary file 1 [file web_material_wrag207.docx]

**Supplemental Material**

**Zoo gut plastispheres enable pathogen escape and adaptation**

Zipei Luo^1^, Yilun Liu^1^, Haimei Wu^1^, Yunmu Xiao^1,2^, Yong Li^1*^, Meizhi Liu^1^, Changchao Li^3^, Dong Zhu^4, 5*^, Ling N. Jin^3,6,7*^, Tao Dong^8^, Wende Yan^1^

^1^ National Engineering Laboratory of Applied Technology for Forestry & Ecology in South China, and Laboratory of Urban Forest Ecology of Hunan Province; Department of Ecology and Environment, Central South University of Forestry and Technology, Changsha, Hunan Province 410004, China

^2^ Key Laboratory of Food & Environment & Drug Monitoring and Testing of Universities in Hunan Province, Hunan Police Academy, Changsha 410138, China

^3^ Department of Civil and Environmental Engineering, The Hong Kong Polytechnic University, Kowloon, Hong Kong, China

^4^ State Key Laboratory of Regional and Urban Ecology, Ningbo Observation and Research Station, Institute of Urban Environment, Chinese Academy of Sciences, Xiamen 361021, China

^5^ Zhejiang Key Laboratory of Pollution Control for Port-Petrochemical Industry, CAS Haixi Industrial Technology Innovation Center in Beilun, Ningbo 315830, China

^6^ Department of Health Technology and Informatics; Research Centre for Nature-based Urban Infrastructure Solutions; Research Centre for Future Food; Mental Health Research Centre, The Hong Kong Polytechnic University, Kowloon, Hong Kong, China

^7^ State Key Laboratory of Marine Environmental Health, City University of Hong Kong, Kowloon, Hong Kong, China

^8^ Department of Immunology and Microbiology, School of Life Sciences, Southern University of Science and Technology, Shenzhen 518055, China

* Corresponding author: liyong_0606@126.com (Yong Li); ling.jin@polyu.edu.hk (Ling N. Jin); dzhu@iue.ac.cn (Dong Zhu)

**This file includes:**

[Supplemental figures	4](#_Toc236588861)

[Figure S1. Experimental design and overall framework.	4](#_Toc236588862)

[Figure S2. Schematic overview of the Twin-SHIME system, consisting of two identical SHIME units (Units 1 and 2).	5](#_Toc236588863)

[Figure S3. Human–animal interaction patterns influence microplastic characteristics in the animal gut environment.	6](#_Toc236588864)

[Figure S4. Polymer types of microplastics in the animal gut.	7](#_Toc236588865)

[Figure S5. Scanning electron microscopy (SEM) images of biofilm and control microplastic surfaces across five polymer types.	8](#_Toc236588866)

[Figure S6. Metagenomic taxonomic profiles of gut-simulation and MP-associated plastisphere communities at the domain level.	9](#_Toc236588867)

[Figure S7. Principal coordinates analyses (PCoA) and PERMANOVA of microbial community structure in animal gut and plastisphere samples. The left ordination compares gut and plastisphere samples, and the right ordination compares Tiger and Elephant samples.	10](#_Toc236588868)

[Figure S8. Antibiotic resistance gene (ARG) composition by antibiotic class in gut and plastisphere samples from Tiger and Elephant.	11](#_Toc236588869)

[Figure S9. Twenty most abundant antibiotic resistance genes (ARGs) in Tiger and Elephant gut and plastisphere samples. Values were log-transformed.	12](#_Toc236588870)

[Figure S10. Detected pathogen counts in gut and plastisphere samples from Tiger and Elephant systems.	13](#_Toc236588871)

[Figure S11. Correlation networks linking virulence factor genes (VFGs) and bacterial communities in Tiger samples. Green and pink nodes denote VFGs and bacteria, respectively; pink and green edges indicate positive and negative correlations, respectively.	14](#_Toc236588872)

[Figure S12. Correlation networks linking virulence factor genes (VFGs) and bacterial communities in Elephant samples. Green and pink nodes denote VFGs and bacteria, respectively; pink and green edges indicate positive and negative correlations, respectively.	15](#_Toc236588873)

[Figure S13. Ten most abundant level 3 KEGG pathways in gut and plastisphere samples from Tiger and Elephant systems.	16](#_Toc236588874)

[Figure S14. Habitat niche breadths of bacterial secretion system genes in gut and plastisphere communities from Tiger and Elephant systems. Significance was assessed with Wilcoxon rank-sum tests	17](#_Toc236588875)

[Figure S15. Bacterial activity and inactivation rates on non-microplastic and microplastic surfaces during aqueous environments.	18](#_Toc236588876)

[Figure S16. Polysaccharide (A) and protein (B) contents on microplastic and non-microplastic surfaces during aqueous exposure.	19](#_Toc236588877)

[Figure S17. Chao1 richness estimates for bacterial communities on MPs and non-MPs during aqueous exposure.	20](#_Toc236588878)

[Figure S18. Level 3 KEGG pathways profiles of bacterial communities on MPs and non-MPs in animal feces during aqueous exposure.	21](#_Toc236588879)

[Supplemental tables	22](#_Toc236588880)

[Table S1. Comparison of microplastic abundance and size distribution in feces from zoo and wild animals of the same species	22](#_Toc236588881)

[Table S2. Summary of the metagenomic sequencing, assembly, and gene prediction for the DNA samples extracted from Tiger and Elephant samples.	23](#_Toc236588882)

Supplemental figures

# Figure S1. Experimental design and overall framework.

Figure S2. Schematic overview of the Twin-SHIME system, consisting of two identical SHIME units (Units 1 and 2).

Liquid feed (A) or liquid feed supplemented with microplastics (B), together with pancreatic juice, was introduced into the stomach and small intestinal compartments, respectively. After a 4-hour retention period, the effluent passed sequentially through the ascending (ASC), transverse (TR), and descending (DES) colon compartments, each maintained under defined pH and hydraulic retention conditions. The colonic compartments were inoculated with fecal microbiota from either Tiger or Elephant, and anoxic conditions were maintained by continuous N₂ flushing with constant agitation at 36.5 °C.

Figure S3. Human–animal interaction patterns influence microplastic characteristics in the animal gut environment.

Unconstrained principal coordinates analysis (PCoA) and PERMANOVA indicated an association between microplastic characteristics in the animal gut environment and patterns of human-animal interaction.

# Figure S4. Polymer types of microplastics in the animal gut.

Figure S5. Scanning electron microscopy (SEM) images of biofilm and control microplastic surfaces across five polymer types.

A–E (Tiger) and K–O (Elephant). Biofilms on the MPs. F–J (Tiger), P–T (Elephant) and U1–Y1; U2–Y2 (MPs incubated without gut inoculum). Diverse microbes observed in the biofilms.

Figure S6. Metagenomic taxonomic profiles of gut-simulation and MP-associated plastisphere communities at the domain level. The NR/DIAMOND-based annotation (A) and the MetaPhlAn-based (B) validation yielded consistent patterns dominated by Bacteria.

# Figure S7. Principal coordinates analyses (PCoA) and PERMANOVA of microbial community structure in animal gut and plastisphere samples. The left ordination compares gut and plastisphere samples, and the right ordination compares Tiger and Elephant samples.

# Figure S8. Antibiotic resistance gene (ARG) composition by antibiotic class in gut and plastisphere samples from Tiger and Elephant.

# Figure S9. Twenty most abundant antibiotic resistance genes (ARGs) in Tiger and Elephant gut and plastisphere samples. Values were log-transformed.

# Figure S10. Detected pathogen counts in gut and plastisphere samples from Tiger and Elephant systems.

# Figure S11. Correlation networks linking virulence factor genes (VFGs) and bacterial communities in Tiger samples. Green and pink nodes denote VFGs and bacteria, respectively; pink and green edges indicate positive and negative correlations, respectively.

# Figure S12. Correlation networks linking virulence factor genes (VFGs) and bacterial communities in Elephant samples. Green and pink nodes denote VFGs and bacteria, respectively; pink and green edges indicate positive and negative correlations, respectively.

# Figure S13. Ten most abundant level 3 KEGG pathways in gut and plastisphere samples from Tiger and Elephant systems.

Figure S14. Habitat niche breadths of bacterial secretion system genes in gut and plastisphere communities from Tiger and Elephant systems. Significance was assessed with Wilcoxon rank-sum tests (**P* < 0.05; ***P* < 0.01; and ****P* < 0.001).

Figure S15. Bacterial activity and inactivation rates on non-microplastic and microplastic surfaces during aqueous environments.

Bars indicate bacterial activity (×10³), and lines indicate bacterial inactivation rate. MDR denotes the mean decline rate in bacterial activity from day 0 through day 35.

# Figure S16. Polysaccharide (A) and protein (B) contents on microplastic and non-microplastic surfaces during aqueous exposure.

# Figure S17. Chao1 richness estimates for bacterial communities on MPs and non-MPs during aqueous exposure.

Figure S18. Level 3 KEGG pathways profiles of bacterial communities on MPs and non-MPs in animal feces during aqueous exposure. Asterisks (*) indicate significant differences between two groups. (Wilcoxon rank-sum test).

Supplemental tables

# Table S1. Comparison of microplastic abundance and size distribution in feces from zoo and wild animals of the same species

| Type | Location | Species | Average MP size (µm) | Mean abundance (items/g d.w.) | Reference |
| --- | --- | --- | --- | --- | --- |
| Wild | China | Amur tiger | 47.82 | 21.37 | [1] |
| Zoo | China | Siberian tiger | 58.30 | 212 | This study |
|  |  | Bengal tiger | 60.2 | 125 | This study |
|  |  | Jaguar | 51.08 | 124 | This study |
| Wild | China | Sika deer | 50.96 | 17.41 | [1] |
|  |  | Roe deer | 45.96 | 20.51 | [1] |
| Zoo | China | Sika deer | 61.03 | 181 | This study |
| Wild  Zoo | Thailand  China | Asian elephant  Elephant | -  52.33 | 0.0182  188 | [2]  This study |

# Table S2. Summary of the metagenomic sequencing, assembly, and gene prediction for the DNA samples extracted from Tiger and Elephant samples.

| Sample | Raw reads | Raw bases (Gbp) | Clean reads | Clean bases (Gbp) | N50 (bp) | N90 (bp) | ORFs | Mean ORF length (bp) |
| --- | --- | --- | --- | --- | --- | --- | --- | --- |
| Tiger |  |  |  |  |  |  |  |  |
| Gut | 69896732 | 10.5 | 59867164 | 8.9 | 1970.0 | 634.7 | 212214 | 651.2 |
| PET | 108838961 | 16.3 | 76925877 | 11.4 | 2116.3 | 630.7 | 251596 | 671.1 |
| PS | 123251804 | 18.5 | 67165708 | 10.0 | 2373.3 | 638.7 | 244497 | 668.8 |
| PVC | 74158850 | 11.1 | 49141072 | 7.3 | 2236.7 | 635.7 | 192726 | 677.0 |
| PE | 77054112 | 11.6 | 50053807 | 7.4 | 2436.0 | 631.3 | 238506 | 666.8 |
| PP | 94066454 | 14.1 | 63436790 | 9.5 | 1854.3 | 620.0 | 234444 | 647.6 |
| Elephant |  |  |  |  |  |  |  |  |
| Gut | 76133417 | 11.4 | 60000407 | 8.9 | 2503.3 | 652.0 | 199088 | 673.0 |
| PET | 72988785 | 11.0 | 54505912 | 8.1 | 2018.7 | 629.0 | 228094 | 654.5 |
| PS | 66797894 | 10.0 | 49502227 | 7.4 | 1832.3 | 620.7 | 161168 | 658.7 |
| PVC | 91814939 | 13.8 | 61856294 | 9.2 | 2292.0 | 634.3 | 221727 | 663.5 |
| PE | 90670519 | 13.6 | 52864972 | 7.8 | 2362.7 | 635.0 | 194603 | 673.6 |
| PP | 84530370 | 12.7 | 48112637 | 7.1 | 2431.7 | 641.3 | 197618 | 672.6 |

Note: Values are presented as the means of three biological replicates per treatment (*n* = 3). Read and ORF counts were rounded to the nearest whole number, whereas base yields, N50, N90, and mean ORF length were reported to one decimal place.

**References**

1. Huang, Z., Liu, D., Cheng, W. et al. Microplastics in the Amur tiger's habitat: Occurrence, characteristics, and risk assessment. *J. Hazard. Mater.* **493**, 138380 (2025).

2. Teampanpong, J. & Duengkae, P. Using feces to indicate plastic pollution in terrestrial vertebrate species in western Thailand. *PeerJ* **12**, e17596 (2024).
